# Implementation of a program for type 2 diabetes based on the Chronic Care Model in a hospital-centered health care system: "the Belgian experience"

**DOI:** 10.1186/1472-6963-9-152

**Published:** 2009-08-23

**Authors:** Patricia Sunaert, Hilde Bastiaens, Luc Feyen, Boris Snauwaert, Frank Nobels, Johan Wens, Etienne Vermeire, Paul Van Royen, Jan De Maeseneer, An De Sutter, Sara Willems

**Affiliations:** 1 Department of General Practice and Primary Health Care, Ghent University, Belgium; 2 Department of General Practice, Interdisciplinary Healthcare and Geriatrics, Antwerp University, Belgium; 3 Department of Endocrinology, OLV-Hospital, Aalst, Belgium

## Abstract

**Background:**

Most research publications on Chronic Care Model (CCM) implementation originate from organizations or countries with a well-structured primary health care system. Information about efforts made in countries with a less well-organized primary health care system is scarce. In 2003, the Belgian National Institute for Health and Disability Insurance commissioned a pilot study to explore how care for type 2 diabetes patients could be organized in a more efficient way in the Belgian healthcare setting, a setting where the organisational framework for chronic care is mainly hospital-centered.

**Methods:**

Process evaluation of an action research project (2003–2007) guided by the CCM in a well-defined geographical area with 76,826 inhabitants and an estimated number of 2,300 type 2 diabetes patients. In consultation with the region a program for type 2 diabetes patients was developed. The degree of implementation of the CCM in the region was assessed using the Assessment of Chronic Illness Care survey (ACIC). A multimethod approach was used to evaluate the implementation process. The resulting data were triangulated in order to identify the main facilitators and barriers encountered during the implementation process.

**Results:**

The overall ACIC score improved from 1.45 (limited support) at the start of the study to 5.5 (basic support) at the end of the study. The establishment of a local steering group and the appointment of a program manager were crucial steps in strengthening primary care. The willingness of a group of well-trained and motivated care providers to invest in quality improvement was an important facilitator. Important barriers were the complexity of the intervention, the lack of quality data, inadequate information technology support, the lack of commitment procedures and the uncertainty about sustainable funding.

**Conclusion:**

Guided by the CCM, this study highlights the opportunities and the bottlenecks for adapting chronic care delivery in a primary care system with limited structure. The study succeeded in achieving a considerable improvement of the overall support for diabetes patients but further improvement requires a shift towards system thinking among policy makers. Currently primary care providers lack the opportunities to take up full responsibility for chronic care.

**Trial registration number:**

ClinicalTrials.gov Identifier: NCT00824499

## Background

Chronic diseases have a growing impact on society and on health care expenditure. Many countries are currently adapting their healthcare system to tackle this burden and often the Chronic Care Model (CCM) is used as a conceptual framework [1]. This model embodies the widespread recognition that implementation of evidence requires whole system change implicating the individual, the structure and the organization [2]. The model respects the role of the primary care physician and emphasises the role of the personal physician, supported by an integrated team. To assure high quality care the CCM recommends organizational changes in six areas: organization of health care, community linkages, delivery system design, self-management support, decision support and clinical information systems [3].

Most of the evidence concerning the components of the model is gathered in the standardized settings of randomised controlled trials. Translating this evidence into practice is not obvious and it is known that the context in which the model is introduced will influence the success of the implementation [4]. The CCM tends to be implemented more successfully in countries with a well-structured primary health care system [5-7]. Information about efforts made in countries where the primary health care system is less well-organized, is scarce [8].

Facing the rising numbers of chronic disease patients and the related burden on the health care system, the Belgian National Institute for Sickness and Disability Insurance commissioned a pilot study exploring how care for type 2 diabetes patients could be organized in a more efficient way in the Belgian health care setting, where the organizational framework for chronic care is mainly hospital-centered. Although the study focused primarily on the care for type 2 diabetes patients, it was the intention to develop an approach, applicable to other chronic diseases in primary care.

The main characteristics of the Belgian health care system are summarized in table 1 and [9,10]. Equity and freedom of choice are high priorities for health policy leaders. There is no clearly defined gatekeeper function in place; every citizen has free access to medical specialists and hospital care, even as the first point of contact with the health system. Most of the care providers work as independent self-employed health professionals. The patient pays directly to the care provider and is reimbursed by the sickness fund. Most services are reimbursed at a rate of 75%. In general, patient satisfaction with the health care system is high [11].

**Table 1:** Main characteristics of the Belgian health care system

• Financing basis of public health is contribution and tax based
• Health expenditure amounts to 9.3% of gross domestic product (GDP)
• Health care expenditure per capita is 2922 (in US$ PPP)
• Coverage of population by public health is almost universal (compulsory)
• Out of pocket payments (proportion of total health expenditure) equals 23%
• Payment of GP is mainly fee-for-service with a limited capitation fee for registered patients
• GP to population ratio 1:909
• GP has no gatekeeper function; voluntary patient registration with a GP (since 1999)
• Approximately 70% of the GPs are working in single-handed practices
• About 66% of the GPs are using an electronic medical record (EMR)

To date, efforts to support patients with chronic diseases are mainly hospital-centered. Concerning type 2 diabetes, patients treated with diet and oral medication are usually seen in primary care, although many of them also receive secondary and/or tertiary care. Teams in primary care are usually flexible and loose networks of single-handed care providers. Structured self-management support programs are not available in primary care. Once insulin is required, most of the patients are referred to a diabetes centre in the hospital. In these centres, patients can consult a multidisciplinary team (diabetologist, diabetes educator, dietician) and have the opportunity to receive expert advice from other disciplines.

This paper reports on a 4-year action research project (1^st ^July 2003 – 30^th ^June 2007) conducted in a well-defined geographical area [12]. Guided by the CCM a regional program has been developed in order to improve care delivery for type 2 diabetes patients in primary care. The challenge of the study was not to prove the effectiveness of the CCM but rather to explore how components of the CCM can be implemented in a primary health care system with limited structure.

The paper addresses the following questions:

1. To what extent could the different components of the CCM be implemented in the region?

2. Which important facilitators and barriers were encountered during the implementation process?

## Methods

### Nature of the intervention

In order to explore ways to adapt the primary health care system to a more chronic care-centered system we chose the methodology of action research [13]. The purpose was to develop a program, on the one hand based on the needs and the opportunities in the region and on the other hand on the evidence concerning provision of effective chronic care. The successive steps of the model for Accelerating Improvement, a scientific method used for action-oriented learning, were followed and an implementation strategy was developed based on the current evidence on successful implementation of care innovation [14,15]. Key features of the implementation strategy were: regional approach, commitment of senior leaders, facilitation by a program manager, bottom-up approach, priority setting, information campaign targeting care providers and patients. The study was conducted in the region by researchers (PS, HB, LF, BS) from two academic primary health care departments in Belgium. The research team worked in close cooperation with senior leaders in the region, primarily the chairman of the GP's association, the chairman of the regional home care organization and the specialists from both regional hospitals. In 2003, a written survey, assessing the strengths and the weaknesses of the organization of diabetes care in the region, was organized by the research team among all care providers involved in diabetes care. Based on the results of the survey, priorities for change were defined in consultation with the local care providers. The CCM was used as framework for planning the interventions. The main components of the intervention were: development and implementation of an interdisciplinary care protocol (delivery system design), development and implementation of a structured education program for type 2 patients on diet or oral therapy (self-management support), establishment of a local steering group and the appointment of a program manager (organization of the healthcare delivery system), provider education (decision support) and regional audit (clinical information systems). The two first components were linked directly to the needs expressed by the region; the latter three were related to the crucial preconditions for the implementation of care innovation. The components of the intervention were introduced progressively in the region.

### Study setting

The study was conducted in the region of Aalst (Flanders, Belgium), a region with 76,826 inhabitants and an estimated number of 2,300 type 2 diabetes patients. In 2004 83 general practitioners (GPs) were active in the area, corresponding with a GP to population ratio of 1:925. Seventy percent of the GPs work in a single-handed practice, mostly without any support staff. One dietician with accreditation for diabetes, five podiatrists with accreditation for diabetes, 46 pharmacists and 90 nurses (5 with diabetes accreditation) are working in the primary care setting. The region of Aalst has two hospitals, both of which have a registered diabetes centre. The intervention population included all type 2 diabetes patients and all care providers involved in diabetes care in the defined area.

### Evaluation approach

In order to answer the first research question, the Assessment of Chronic Illness Care (ACIC, Version 3.0) survey was used to evaluate the degree of implementation of the CCM in the region. The ACIC survey has been developed to evaluate chronic illness care in different settings and for one specific condition at a time [16,17]. We used the survey to evaluate the support for diabetes care in a well-defined region. In the ACIC, the highest score ("11") on any individual item, subscale, or the overall score (an average of the six subscale scores) indicates optimal support for chronic illness. The lowest possible score on any given item or subscale is a "0", which corresponds to limited support for chronic illness care. In order to increase standardization and reproducibility, prompts are formulated for each item. To complete the survey, we followed the directions described in the manual available on the Improving Chronic Illness Care (ICIC) website [18]. A precondition to complete the survey is that one needs to be familiar with the system. In our case, four members of the research team (PS, HB, LF, BS) and two members of the local team (MA, FN) took part in the scoring procedure. All six were members of the local steering group and had been involved in the study from the beginning in 2003. They also had access to all of the information collected during the study. The survey was completed at the end of the study period to describe the degree of implementation of the CCM in 2003 (beginning of the study period) and in 2007 (end of the study period). First, each of the participants in the scoring procedure completed a separate form. Next, a meeting was organized in order to reach consensus about the scores. During this meeting, a qualitative justification for each score was obtained in order to enhance the validity of the scoring. The meeting lasted 2 hours. A report of the meeting was sent for approval to all participants in the scoring procedure and was accepted. Finally, the qualitative justification for each item was also discussed in the entire steering group to validate the findings. No adjustments were needed.

In order to answer the second research question a document analysis was performed by one of the researchers (PS) [19]. Meeting reports collected during the 4-year study period were analyzed in order to identify the main facilitators and barriers encountered during the implementation period. A set of 113 documents was analysed (31 study group meeting reports, 5 interdisciplinary group meeting reports, 2 meeting reports with the local quality group leaders, 37 steering group meeting reports and 38 research group meeting reports). The reports were analyzed (familiarization, coding into themes, applying the framework to the data, interpretation) with a theoretical framework related to the work of Grol and Wensing as background. This framework links the barriers and facilitators to the characteristics of the intervention program, the professionals, the patients, the social context, the economic, administrative and organisational context and the implementation method [20,21]. Analysis was done with the assistance of a qualitative data analysis software program (NVivo 2.0). To form a more complete picture of the issue, the results of the document analysis were subsequently triangulated with the results of the analysis of other data sources collected during the study (surveys, individual interviews, focus group interviews; table 2) [22,23]. All data were collected and analysed according standardized procedures [24]. A trained interviewer (MV), psychologist and researcher, performed the individual interviews with patients and GPs. All interviews (individual, focus group) were audio taped and transcribed. Two researchers performed the coding independently (PS and MV (non participation, insulin therapy); HB and LS (individual education program); PS and LF (focus groups)). Three researchers (PS, HB, BS) performed the triangulation process separately guided by the triangulation protocol of Farmer (sorting, convergence coding, convergence assessment, completeness assessment) [25]. First, the findings were analyzed independently using the framework of Grol and Wensing as background. Next, consensus on the aggregated data was reached through discussion (researcher comparison). There was a high level of agreement between the three researchers concerning the most important supporting and hindering factors in the implementation process. Finally, a summary of the triangulated results was sent for review to the members of the research team and the members of the local steering group (feedback). They agreed with the results.

**Table 2:** Data sources available for triangulation

Evaluation of the needs of the region concerning the diabetes care delivery	-Structured questionnaire among all care providers involved in type 2 diabetes care assessing the strengths and the needs concerning diabetes care delivery in the region. Response rate: 314 questionnaires. GPs (49), specialists (5), home care (144), nurses (70), podiatrists (6), pharmacists (25), dieticians (5), eye specialists (10). Period: 2003–2004.
Evaluation of the complex intervention	-Continuous registration of the participation rate in the different components of the intervention.
	
	-Semi-structured interviews among GPs who did not or only moderately participated in the program. Purposive sample of 20 GPs. Period: 2007
	
	-Structured questionnaire among GPs concerning participation in the project and organization of chronic care in 2007. Response rate: 61 questionnaires. Period: 2007
	
	-Structured telephonic questionnaire among pharmacists evaluating their participation in the project and the implementation of the medication record. Response: 46. Period: 2005 and 2006
	
	-Document analysis of 113 meeting reports (31 study group meetings, 5 interdisciplinary group meeting, 2 meeting reports with the local quality group leaders, 37 steering group meetings and 38 research group meetings) exploring the main facilitators and barriers during the implementation process. Period: 2007
	
	-Medical record audit by GPs in order to assess the quality of diabetes care. Information available from 20% of the estimated type 2 diabetes population in 2004, from 14% in 2006.

Evaluation of the regional support structure (organization of health care)	-Semi-structured questionnaire concerning the functioning of the regional support structure among the members of the local steering group not belonging to the research team. Response rate: 4 written surveys in 2006, 8 written surveys in 2007.
	
	-Semi-structured questionnaire concerning the task profile of a program manager. Response: 1 questionnaire. Period: 2007.

Evaluation of the education program (self-management support)	-Semi-structured interviews among patients who participated in the education program. Purposive sample of 22 patients. Period: 2006–2007
	
	-Semi-structured interviews among GPs who referred patients on a regular basis. Purposive sample of 15 GPs. Period: 2006–2007
	
	-Semi-structured written questionnaire concerning the task profile of the educator: 1 questionnaire. Period: 2007.

Evaluation of the support program for insulin initiation (delivery system design)	-Structured written questionnaire among GPs evaluating potential barriers for insulin initiation. Response rate: 37 questionnaires. Period: 2005
	
	-Focus group interview with GPs who referred patients for insulin initiation. 2 focus groups with 13 GPs. Period: 2006
	
	-Semi-structured interviews with patients who started insulin therapy. Purposive sample of 10 patients. Period: 2007
	
	-Record analysis of patients referred for insulin initiation. 55 patient records. Period: 2007

Evaluation of the quality monitoring project (clinical information systems)	-Structured written questionnaire among GPs evaluating barriers for participating in the quality monitoring project. Response rate: 45 questionnaires. Period: 2005

The study was approved by the Ethical Committee of the University of Ghent (Approval number 2004/253) and the Ethical Committee of the University of Antwerp (Approval number 12/07/2004).

## Results

Results are presented in two parts. The first part describes the degree of implementation of the CCM in the region based on the ACIC scores. The second part reports on the facilitators and barriers encountered during the implementation process.

### Implementation of the CCM components

#### ACIC scores

Table 3 gives an overview of the ACIC scores for the region (individual items, subscales, overall score) in 2003 and in 2007. An overall ACIC score of 1.45 in 2003 reflected the limited support for chronic care delivery in the region at the start of the project. In 2007, the overall score had increased to 5.5. The scores of all components increased, although those for delivery system design and clinical information systems changed the least. A justification for each individual item score can be consulted in the Additional file (see Additional file 1). The main actions implemented in the region to improve care delivery for type 2 diabetes patients are described below. The actions are classified under the CCM component they relate to.

**Table 3:** Assessment of Chronic Illness Care (ACIC) for type 2 diabetes patients  in the region

**ACIC components**	**Score 2003**	**Score 2007**	**Max score**
**Part 1: Organization of the Healthcare Delivery System**	**2,7**	**7**	**66/6**

-Overall organizational leadership in chronic illness care	2	7	11

-Organizational goals for chronic care	1	7	11

-Improvement strategy for chronic illness care	1	9	11

-Incentives and regulations for chronic illness care	0	0	11

-Senior leaders	6	10	11

-Benefits	6	9	11

**Part 2: Community Linkages**	**0,3**	**5,7**	**33/3**

-Linking patients to outside resources	1	5	11

-Partnership with community organizations	0	6	11

-Regional health plans	0	6	11

**Part 3: Practice level**			

**Part 3a: Self-management support**	**1,3**	**6,3**	**44/4**

-Assessment and documentation of self-management needs and activities	3	6	11

-Self-management support	1	6	11

-Addressing concerns of patients and families	1	6	11

-Effective behaviour change interventions and peer support	0	7	11

**Part 3b: Decision support**	**2,5**	**7,3**	**44/4**

-Evidence-based guidelines	3	8	11

-Involvement of specialists in improving primary care	2	9	11

-Provider education for chronic illness care	2	5	11

-Informing patients about guidelines	3	7	11

**Part 3c: Delivery System Design**	**1,3**	**4,5**	**66/6**

-Practice team functioning	0	5	11

-Practice team leadership	1	9	11

-Appointment system	1	2	11

-Follow-up	2	3	11

-Planned visits for chronic illness care	0	0	11

-Continuity of care	4	8	11

**Part 3d: Clinical information systems**	**0,6**	**2,4**	**55/5**

-List of patients with specific conditions	1	2	11

-Reminders tot providers	0	0	11

-Feedback	0	4	11

-Information about relevant subgroups of patients needing services	0	0	11

-Patient treatment plans	2	6	11

**Overall ACIC score**	**1,45**	**5,5**	**11**

The ACIC is organized such that the highest score ("11") on any individual item, subscale or the overall score indicates optimal support for chronic illness.

Between "0" and "2" = limited support for chronic illness care

Between "3" and "5" = basic support for chronic illness care

Between "6" and "8" = reasonably good support for chronic illness care

Between "9" and "11" = fully developed chronic illness care

#### Subscale 1: Organization of healthcare delivery system

Until 2003 there was little or no regional coordination regarding chronic care delivery. The project succeeded in establishing a regional coordinated program for type 2 diabetes patients. In 2003, a local steering group was established including two GPs, two specialists and four members of the research team. In July 2004, a program manager and two diabetes educators were employed and joined the steering group. The steering group provided three kinds of expertise (system leadership, technical expertise, day-to-day leadership) and met on a monthly basis. In 2006, a podiatrist and a pharmacist joined the steering group, the representation of the research team in the steering group was then reduced to one single member. In 2004 the program manager installed study groups for the different disciplines working with diabetes patients (GPs, specialists, pharmacists, dieticians, podiatrists, nurses, home care). Forty-one care providers participated in the study groups. Participants were recruited through their professional organizations. The purpose of the installation of the study groups was to engage the local health care providers in the innovation process. The study groups were asked to formulate discipline-specific opportunities, needs and concerns related to the care for diabetes patients. Each study group delivered a document with a report of the meetings and priorities for change. Subsequently, two representatives of each discipline formed an interdisciplinary study group. In consultation with the research team this group developed an interdisciplinary care protocol for type 2 diabetes based on the national diabetes guideline and tailored to the needs of the region [26,27]. During the project the interdisciplinary study group met at least once a year. Priority setting for change was done in consultation with this group. The study groups remained active and were consulted whenever new initiatives were considered. The program manager played an important role in the day-to-day leadership of the project and in the facilitation of the implementation process [28]. The local team was operating from a central location in the region. Financial resources were supplied by project funding. These financial resources were mainly needed for the wages of the educators and the program manager, the expenses for hiring a central location in the region and the costs of the information campaigns. Usual care was financed like before.

#### Subscale 2: Community linkages

During the project, partnerships with different community organisations were created. From the start there was regular contact with the local diabetes patients' organization. Information sessions for patients were mostly organized in collaboration. A regional "patient day" for type 2 diabetes patients was organized jointly in January 2006. As the project progressed, contacts with socio-cultural partners, e.g. senior organizations, increased. The educators were also consulted to provide training sessions for the staff of nursing homes in the region.

#### Subscale 3a: Self-management support

In October 2004, an individual education program for type 2 diabetes patients was launched in the region. The education program was based on the empowerment theory and was developed in consultation with the study groups [29]. Two diabetes educators were engaged. The program was open for all patients on diet or oral medication and was free of charge. Disabled people could ask for a home visit. Referral from the GP was obligatory to enter the education program. This way we wanted to stress on the link between the education program and the regular health care people received from their GP. According to the interdisciplinary care protocol, GPs had an important role in identifying and motivating suitable patients for self-management support. Two years after the introduction of the program in the region 70% of the GPs had referred at least one patient to the program. The percentage of GPs referring patients to the education program increased progressively. The progress in adoption of the new service by GPs was comparable with figures in other care innovation projects [30]. About 20% of the estimated number of diabetes patients on diet or oral therapy in the region attended the program. Patients were informed on a regular basis about the education program through announcements in the local newspapers. In June 2005, a small-scale pilot, evaluating the feasibility of a group-based program was started in the region [31].

#### Subscale 3b: Decision support

A national guideline on diabetes care was available and was used to guide the interventions. During the project interdisciplinary training sessions focusing on the implementation of the protocol were organized. Sixty percent of the GPs participated in at least one of the training sessions. The degree of involvement of the specialists in educational support for primary care increased during the study.

#### Subscale 3c: Delivery system design

The interdisciplinary care protocol included clear task descriptions with respect for the role of the GP and guidelines for interdisciplinary communication. Case management for the initiation of insulin therapy in primary care was organized in collaboration with the specialists. Patients could be referred from 1 February 2005 onwards. Specialists coached the support program for the initiation of insulin therapy: educators and GPs had the opportunity to consult specialists for advice on the therapy scheme by phone or e-mail. Two years after the introduction of the support program in the region, 30% of the GPs had referred at least one patient to the program.

#### Subscale 3d: Clinical information systems

The lack of systematic data on the quality of care in the region forced us to ask GPs to participate in a quality monitoring project. In September 2004, all GPs were invited to register information about all their patients or, if this was not feasible, about the first 10 patients they met in the following weeks. A standardized registration form, based on the DiabCare registration form was developed in consultation with the study group of GPs [32]. Informed consent from patients was obtained. About half of the GPs (49%) contributed to the registration. As a result, a database with information from approximately 20% of the estimated diabetes population in the region could be set up. In 2005, feedback on the quality of care on a regional level was sent to all GPs and specialists in the region. The objective of the establishment of a database with yearly feedback was abandoned due to the problems encountered in the registration process. In 2006, a follow-up registration focused only on the patients registered in 2004.

### Facilitators and barriers in the implementation process

Table 4 summarizes the main facilitators and barriers encountered during the implementation of the program. Facilitators and barriers were identified on different levels including characteristics of the intervention program, the professionals, the patients, the social context, the economic, administrative and organisational context and the implementation method. The most remarkable facilitators and barriers are described below.

**Table 4:** Main facilitators and barriers encountered during the implementation process

**Characteristics of the intervention program**	
**Facilitators**	**Barriers**

-The program was tailored to the needs expressed by the region. The emphasis was on care coordination and self-management support for patients. -The engagement of a program manager in the region provided the opportunity to strengthen the network of care providers in the region. -The program respected and reinforced the role of primary care in chronic care delivery. When new services were introduced in the region (education program, support program for the initiation of insulin therapy), respect for the role of each discipline and interdisciplinary communication were central points in the way of collaboration.	-The program targeted different components of the CCM. This resulted in a complex intervention. The complexity of the intervention hampered the information campaign and some components of the intervention negatively affected each other. -The program was not clearly defined at the start of the project. It was the intention to develop the program in collaboration with the region. This led to some confusion about the aims of the project among care providers. -Some aspects of the program increased the administrative burden on GPs (regional audit, informed consent procedures).

**Characteristics of the professionals**	

**Facilitators**	**Barriers**

-The project could rely on a group of well trained and motivated care providers from different disciplines to collaborate with the project. -Specialists from both hospitals were prepared to contribute to the project. They were involved in the decision making process in the region through their representation in the steering group.	-Care providers had no tradition of working with an interdisciplinary care protocol. Neither did they had the tradition to commit to agreements made by their representatives. -Care providers expressed their fear of losing patients to the education program. GPs feared to lose control over their patients and also feared loss of income. A nurse's' organization considered education as a job for their nurses working in home care. -The threshold to start insulin therapy was still high among some GPs. Lack of knowledge and fear for hypoglycaemia were the most important barriers. -Some care providers didn't participate in the social network and were difficult to reach in information campaigns.

**Characteristics of the patients**	

**Facilitators**	**Barriers**

-Patients who did participate in the education program, expressed their satisfaction with the content of the program. -Patients who did participate in the education program, considered their GP as the central care provider.	-According to the GPs, some patients were hard to motivate to participate in the education program. -Motivating patients to participate in the education program required extra time during the consultation. - According to the GPs, resistance against starting insulin therapy was still high among patients needing insulin therapy. This resistance disappeared usually once insulin therapy was started.

**Characteristics of the social context**	

**Facilitators**	**Barriers**

-Senior leaders in the region gave their commitment to the project from the start. Their support to the program was visible during the whole study time.	-Care providers were dissatisfied with the current health policy. The fact that the National Institute for Sickness and Disability funded the project retained some care providers from participating in the project. -Participation in the different components of the program increased gradually. Care providers and patients needed time to trust the new initiatives. This was in contrast with the commissioners who expected results in a short time.

**Characteristics of the economic, administrative and organisational context**	

**Facilitators**	**Barriers**

-The government has invested in guideline development. A national guideline on type 2 diabetes was in preparation at the start of the project. The guideline was used to develop the program. -Clear legal national task profiles were available. These profiles were used as a guide for the development of the interdisciplinary care protocol. -There was already some degree of regional organization in place: a network of home care providers (representatives of GPs, nurses and home care) and the professional organizations (GPs, nurses, pharmacists)	-At the start of the project figures about the quality of diabetes care in the region were not available. As a consequence a regional audit was organised although this was not expressed as a priority by the region. -When the project started care providers worked in relative isolation. It was not clear who was involved in diabetes care in the region. There was no central registration point for care providers. -The organizational structures present in the region at the start of the project had only limited authority and lacked adequate financial support. -Most GPs had no support staff in practice. -The continuation of the program was unsure. The commissioners hesitated to extend funding after the study period. This uncertainty negatively influenced the participation of the region and was a constant point of concern in the local steering group during the last year of the study.

**Characteristics of the implementation method**	

**Facilitators**	**Barriers**

-Senior leaders were involved in the project from the start. Their support was made visible through their participation in the local steering group. -The existing network of care providers was used and strengthened. -The program was the result of a consultative process in the region and targeted the needs expressed by the region. -Priorities for change were formulated in consultation with the study groups. -The engagement of a program manager facilitated the implementation of the program in the region. -An information campaign was designed addressing care providers and patients on a regular basis.	-The information campaign at the start of the project was experienced as confusing. This was partly because of the strategy of involving the region in the development of the program, partly because the program included different components. -The program targeted different components of the CCM. As a consequence too many priorities were formulated. -There was no tradition of asking care providers for official commitment to agreements made in the region. The project decided not to break with this principle. -The scientific evaluation of the study was time consuming and interfered with the daily work in the region.

#### Facilitators

The first and most important facilitator was the willingness of a group of well-trained and motivated care providers in the region to invest in quality improvement. Senior leaders agreed from the start to cooperate with the study. Later on the program manager had no problem to find volunteers for the study groups. The preparedness of the region to participate in the study was probably the result of the need the health care providers felt for a more efficient organization of diabetes care in the region.

Second, the appointment of a program manager, supported by a local steering group, created opportunities to strengthen the network of care providers in the region. One of the first visible results of the manager's work was a website with among other things a list of all care providers involved in diabetes care. In a later stage of the project, the program manager facilitated the process of development and implementation of the care protocol.

Third, involving the regional health care providers in the development of the program resulted in a better understanding of the needs of the region and subsequently contributed to the appropriateness of the program. The program respected and reinforced the role of the GP in diabetes care. GPs who referred patients to the education program appreciated the way of cooperation with the educator. They felt respected and supported in their coordinating role and the fear to "lose the patient" disappeared.

#### Barriers

An important barrier in the implementation process was the complexity of the intervention. The study started in a context where a lot needed to be changed. This resulted in a program with probably too many components. Each component of the intervention required a specific implementation strategy and follow up. This led to some confusion about the aims of the study and further, some components affected each other negatively. For instance, some GPs decided not to cooperate with the project because of the administrative burden caused by the manual registration of quality data.

The involvement of the local actors in the development of the program also had some disadvantages. Some care providers perceived this procedure as a lack of professionalism and preparation of the research team. Others were confused about the opportunities the project could offer them.

The poor organization of primary care was difficult to overcome. The program manager spent a lot of time updating the database with all care providers in the region and bringing the disciplines together in study groups. This was the first time an interdisciplinary protocol was implemented. During the project it was decided that the protocol would be distributed through the provider's organizations without asking for official commitment by their members. This approach led to a rather limited commitment of individual care providers to the project.

Fear of "losing patients" affected the referral rate to the education program. Although it was emphasized that the educator's work would be complementary to the GP's and that collaboration with the GP was a central focus of the project, fear arose among GPs that the patient would be taken away from the general practice. The same problem occurred with one of the nurse's organizations in the region. This fear was, to a large extent, the consequence of former hospital-driven initiatives that did not respect the role of primary care, e.g. the organization of open clinics in secondary care for obesity and osteoporosis. Moreover, the fee-for-service system does not support collaboration.

Due to the lack of software facilities for automatic data extraction, GPs needed to register quality data on a paper form. This was experienced as an immense administrative burden. Since most practices lack administrative support, the research team proposed to provide assistance in the registration procedure but none of the GPs made use of this offer.

Finally the uncertainty about the future of the program was a major disturbing factor. At the end of the study it became difficult to motivate care providers to participate in a process that maybe wouldn't continue. For instance the referral rate of patients to the education program decreased each time doubt rose about the continuation of the project. The uncertainty about sustainable funding also preoccupied the members of the steering group. They were convinced of the value of the program and had to look for alternative resources. In the last year of the project the search for alternative funding was a dominant theme in the monthly meetings and new initiatives in the region were postponed.

## Discussion

We found that an action research project with the intention of implementing a program based on the CCM in a region with a weak primary health care system succeeded in improving the overall support for diabetes care from a limited to a basic level. Although this was a considerable improvement, it means that there is still a long way to go. During the project we drew some important lessons concerning the implementation strategy and the context in which we worked. This context-specific understanding of the factors that facilitated or hindered the implementation process is crucial for further initiatives in the Belgian context but may be also helpful for other countries struggling to implement the CCM.

### Strengths and limits of the study

An important strength of the study is that it gives a realistic view on the feasibility of implementing a program based on the CCM in a primary health care system with limited structure. The methodology of action research enabled us to explore, in consultation with a whole region, the opportunities and the barriers for adapting the current system to a more accurate chronic care organisation. By targeting the whole region and not only the motivated care providers and patients the study gave a realistic view on the willingness of the region to participate in the project. This approach was chosen in order to avoid a situation where the pilot succeeds, but the conditions under which it succeeds are so different from the norm that it possibly cannot propagate [33]. In addition, the study period was long enough to evaluate the sustainability of the changes introduced in the region.

The CCM and the ACIC survey were used as a guide to set out priorities for change and to plan the interventions. The ACIC survey was completed at the end of the study period to track the progress in chronic care delivery. We are aware that this procedure had potential for bias. The scoring team was strongly involved in the project and possibly tended to give higher scores in 2007 in order to favour the results of the intervention. But we think that we were able to reduce the risk for bias to a minimum by adding a supplementary step in the scoring procedure, namely by adding a qualitative justification for each item score. This step makes the scoring transparent and verifiable. The scoring team had access to all the information collected during the project, among which the description of the system in 2003. Neither the researchers, nor the local team would take direct benefit from higher scores. The focus of the study was essentially explorative, looking for ways to adapt the system to chronic care delivery.

We are currently not able to give information on the relation between the introduction of CCM elements in the region and indicators of quality of care. We did a lot of effort to built up a quality monitoring system in the region but, as mentioned before, this initiative was hampered by the administrative burden it caused amongst the participating GPs. The data we collected in a real life setting were useful to guide the process of priority setting but not valid enough to evaluate the effect of the intervention in the region. In a follow-up study we are building up a database using data from the sickness funds and the laboratories in the region. This database will allow us to compare the quality of care between the intervention region and a control region where usual care is continued.

### CCM as a guide in quality improvement

We experienced the CCM model and the related ACIC survey as very useful for evaluation, reflection, and priority setting in chronic care reorganisation. One of the strengths of the CCM is its emphasis on the need for integration of different components targeting quality improvement. For instance, It makes clear that one cannot blame the individual GP for not participating in quality monitoring when he or she does not have the resources (time, IT support and manpower) to do so. Another strength of the model is that it is suitable for different chronic conditions and that most of the components are generic. Some of the changes achieved in this project, like improving the visibility of the local healthcare workforce and having a forum to make task agreements, are also relevant for other chronic conditions. The description of optimal care in the ACIC survey is a useful guide to formulate concrete goals. Yet what is described as 'optimal care' is not always suitable to all care systems. For instance, in a country with relatively small practices, which is the case in our context, a regionally coordinated education program is more feasible than allocating an educator to each practice. Learning from each other how specific components can be translated in different settings can enhance quality improvement efforts. In this view the qualitative justifications we added to the scores are valuable information. The progress in ACIC score needs to be seen in relation to the score at the start of the project. In a system where figures are very low at the starting point, as is the case in our context, there is much more room for improvement than in systems where almost all components are in place. The CCM gives no clear advice on how or in which order of rank the different components need to be implemented. We experienced the model for Accelerating Improvement as a useful guide during the implementation process.

### Setting out priorities for change

In a context where there is room for improvement in most of the components of the CCM, setting out priorities for change is crucial. We chose to define priorities for change in consultation with the local stakeholders, an approach supported by the implementation research literature. We aimed to introduce a program with high relevance for the region and a high chance of sustainability. The strategy of working in a region and exploring opportunities for care innovation in cooperation with the local care providers concerned has shown its benefits in other settings [34]. However, a good balance between a bottom-up and a top-down approach is needed and strategies should be communicated clearly in order to avoid misinterpretation. Defining too many priorities for change is a pitfall in a context where a lot needs to be changed. The program we introduced in the region was complex and, as mentioned before, some of the components negatively affected each other. Looking back, it would have been better to focus on one or two components at a time. On the other hand, the interdependence of the different components of the CCM makes it difficult to introduce the components individually. For example, the introduction of an education program is not feasible without a clear task description for all actors in the region.

### Strengthening the network of care providers

The strategy of introducing provider networks in primary care in order to overcome a lack of coordination and continuity of care has been used in other health care systems, e.g. the health networks in France [35]. The evidence available from research provides only limited support for the intuitive belief in the potential of integration to solve many problems [36]. Although strengthening the primary care network probably does not lead directly to quality improvement, it is a necessary condition to start quality improvement in a region. The implementation of the task agreements was hindered by the absence of a commitment procedure. Respect for the autonomy of patients (free choice of care provider) and of care providers (diagnostic and therapeutic freedom) are highly rated principles in the Belgian health system. In consultation with the region we chose not to break with these principles but future initiatives will have to address this issue.

### Focus on information technology (IT) and quality monitoring

It is clear that improving the current IT system has to be one of the main priorities for the future. About 70% of the GPs in the region were actually using an electronic medical record (EMR) but software packages in primary care lack essential tools: GPs are not able to build a diabetes register or to extract data for quality monitoring, and reminder systems are not in place. This is reflected in the low ACIC score for clinical information systems. Some successful innovation projects started with the implementation of an adequate EMR system and implemented other components of the CCM later on [37]. An adequate IT system will also facilitate continuous evaluation and feedback, essential aspects of quality improvement. Evaluation and feedback generally enhance the implementation of innovation initiatives but in our study this component was more a limiting than an enabling factor. There are some opportunities in the Belgian context to implement a more efficient IT system and a quality monitoring system in the near future. Since 2003, national criteria for software packages in general practice and a labelling procedure are in place. GPs using a labelled software package receive compensation each year [38]. Furthermore, there is already some experience with quality monitoring for diabetes. Since 2001, quality data from diabetes patients treated in secondary care have been monitored. Data handling and bench marking is performed by the Scientific Institute of Public Health [39]. The fact that there are too many different software packages in primary care with insufficient interoperability, will be an obstacle.

### Reinforcing the role of primary care

Further improvement in the results on the ACIC score for chronic care delivery demands a major change in the organization of primary health care. Health policy leaders in Belgium have emphasized since long the pivotal role of primary care in chronic care, but this has been given more "lip service" than implementation. To date, there has been mainly invested in fragmented initiatives mostly targeting individual patients or individual care providers. This has often resulted in poor participation of adopters. The project was the first to use the CCM in the Belgian context. We introduced the model during interim meetings with the commissioners of the study. In this way we repeatedly emphasized the need to integrate future quality improving initiatives in a coherent plan on chronic care reorganization in primary care, shifting the focus from the individual care providers to the system in which they work. The establishment of patient listing, gate keeping and supporting staff, essential preconditions for adequate chronic care organization, requires national policy measures. There have been some initiatives to strengthen the role of the GP in the past. For example, since 1999 patients can register on a voluntary basis in general practice. Registration is accompanied with incentives for patients and GPs; the percentage of registered patients is gradually increasing, amounting to 43% in Flanders in 2006. These initiatives illustrate that there is awareness among health policy leaders that primary care needs to be reinforced but more radical decisions fail tot occur, partly because of the complexity of the decision process in the Belgian context. If health policy leaders want primary care to take up a central role in chronic care, this issue needs to be placed on the agenda again. Several countries have developed a national policy targeting these topics [40,41].

### Sustainability

Although the commissioners seemed convinced about the opportunities of the intervention, funding of the project was (temporarily) discontinued in 2007. The local steering group, convinced of the value of the program, collected the necessary funds to continue the program. At the time of writing the steering group, the study groups and the interdisciplinary study group are still active in the region. Patients can still attend the education program. Continuation funding for the program is still a point of debate in the Committee for Health Care Insurance.

## Conclusion

The study succeeded in achieving a considerable improvement of the overall support for diabetes patients in primary care but there is still a long way to go. Currently primary care providers lack the opportunities to take up full responsibility for chronic care. By using the CCM in reporting the study results we aimed to contribute to a shift towards system thinking among health policy makers, given the fact that most of the quality improvement initiatives are still focusing on individual patients or individual care providers. The involvement of the region in the exploration of ways to improve the care for type 2 diabetes patients was one of the strengths of this study. This approach contributed to the appropriateness of the implemented program and deepens the insights in the opportunities and barriers to adapt chronic care in primary health care systems with limited structure. The study further adds to the knowledge concerning the implementation of innovation in a region. Although we carefully addressed the factors known to facilitate the spread of innovation, we were confronted with several barriers. An important barrier, of which the importance increased during the study, was the insecurity about sustainability of funding. Future pilot studies need to pay attention to the criteria policy makers will use to decide about continuation or discontinuation of funding, before involving a whole region in a quality improvement effort.

## Competing interests

The authors declare that they have no competing interests.

## Authors' contributions

PS, HB, LF, BS, FN, JW, PVR and JDM participated in the development of the study design. PS, HB, BS, FN played a leading role in the management of the field work. PS, HB, LF and BS drafted the manuscript. EV, PVR, JDM, ADS and SW supervised the data collection and analyses. All authors contributed to, have read and approved the final manuscript.

## Pre-publication history

The pre-publication history for this paper can be accessed here:



## Supplementary Material

Additional file 1 **Assessment of Chronic Illness Care (ACIC) for diabetes type 2 patients in the region**. Qualitative justifications for the individual item scores in the ACIC survey. Click here for file
