# Supplementary material for: Implementation of a program for type 2 diabetes based on the Chronic Care Model in a hospital-centered health care system: "the Belgian experience"
Source: BMC Health Serv Res. 2009 Aug 23;9:152. doi: 10.1186/1472-6963-9-152 (PMC2757022; doi:10.1186/1472-6963-9-152)
Supplement: Additional file 1 — Assessment of Chronic Illness Care (ACIC) for diabetes type 2 patients in the region. Qualitative justifications for the individual item scores in the ACIC survey. [file 1472-6963-9-152-S1.doc]

**Additional file 1:**

**Assessment of Chronic Illness Care (ACIC) for diabetes type 2 patients in the region: qualitative justifications for the individual item scores.**

**Part 1: Organization of the Healthcare Delivery System at the meso level.**

Chronic illness management programs can be more effective if the overall system (organization) in which care is provided is oriented and led in a manner that allows for a focus on chronic illness care.

| **Components** | **Level D** | **Level C** | **Level B** | **Level A** |
| --- | --- | --- | --- | --- |
| **Overall Organizational Leadership in Chronic Illness Care**  **Score** | …does not exist or there is a little interest.  0 1 2 | …is reflected in vision statements and business plans, but no resources are specifically earmarked to execute the work.  3 4 5 | …is reflected by senior leadership and specific dedicated resources (dollars and personnel).  6 7 8 | …is part of the system’s long term planning strategy, receive necessary resources, and specific people are held accountable.  9 10 11 |
| **Score 2003:2 Score 2007:7** | | | | |
| -Until 2003 there was little or no regional coordination regarding chronic care delivery.-In 2003, a local steering group was established including two GPs, two specialists and four members of the research team. In July 2004, a program manager and two diabetes educators were employed and joined the steering group. The steering group provided three kinds of expertise (system leadership, technical expertise, day-to-day leadership) and met on a monthly basis. In 2006, a podiatrist and a pharmacist joined the steering group, the representation of the research team was then reduced to one single member.-The program manager was appointed to take up the day-to-day leadership of the project and to facilitate the implementation process.-The program manager installed study groups for the different disciplines working with diabetes patients (GPs, specialists, pharmacists, dieticians, podiatrists, nurses, home care). Out of these study groups an interdisciplinary study group was founded.-During the project the interdisciplinary study group met at least once a year. Priority setting was done in collaboration with this group*.*-The commissioner of the study (National Institute for Health and Disability) provided dedicated resources for coordination and implementation of care innovation during the project. There was no certainty about the continuity of the funding after the project. | | | | |

| **Organizational Goals for Chronic Care**  **Score** | …do not exist or are limited  0 1 2 | …exist but are not actively reviewed.  3 4 5 | …are measurable and reviewed.  6 7 8 | …are measurable, reviewed routinely, and are incorporated into plans for improvement.  9 10 11 |
| --- | --- | --- | --- | --- |
| **Score 2003:1 Score 2007:7** | | | | |
| -An interdisciplinary study group was established in the region. This group developed an interdisciplinary care protocol for type 2 diabetes tailored to the needs of the region (clear task descriptions for each discipline, establishment of an education program for type 2 diabetes patients on diet and/or oral medication, support program for the initiation of insulin therapy in primary care).  -Participation of the region (patients and care providers) was monitored and reviewed.  -Organizational goals were reviewed but not routinely because of the workload. | | | | |

| **Improvement Strategy for Chronic Illness Care**  **Score** | …is ad hoc and not organized or supported consistently.  0 1 2 | …utilizes ad hoc approaches for targeted problems as they emerge.  3 4 5 | …utilizes a proven improvement strategy for targeted problems.  6 7 8 | …includes a proven improvement strategy and uses it proactively in meeting organizational goals.  9 10 11 |
| --- | --- | --- | --- | --- |
| Score 2003:1 Score 2007:9 | | | | |
| -A program manager was appointed to take up the day-to-day leadership of the project and to facilitate the implementation process. The successive steps of the Model for Accelerating Improvement, a scientific method used for action-oriented learning, were followed. An implementation plan was developed taking into account the current evidence on successful implementation of care innovation. Key features of the implementation strategy were: regional approach, commitment of senior leaders, bottom-up approach, priority setting, information campaign targeting patients and care providers. | | | | |

| **Incentives and Regulations for Chronic Illness Care**  **Score** | …are not used to influence clinical performance goals.  0 1 2 | …are used to influence utilization and costs of chronic illness care.  3 4 5 | …are used to support patient care goals.  6 7 8 | …are used to motivate and empower providers to support patient care goals.  9 10 11 |
| --- | --- | --- | --- | --- |
| **Score 2003:0 Score 2007:0** | | | | |
| -Financial incentives were not used to influence clinical performance goals (no target payment). | | | | |

| **Senior Leaders**  **Score** | …discourage enrollment of the chronically ill.  0 1 2 | …do not make improvements to chronic illness care a priority.  3 4 5 | …encourage improvement efforts in chronic care.  6 7 8 | …visibly participate in improvement efforts in chronic care.  9 10 11 |
| --- | --- | --- | --- | --- |
| **Score 2003:6 Score 2007:10** | | | | |
| -In 2003 senior leaders in the region had already a history of engagement in ad hoc quality improvement projects.  -Before starting the project, commitment was asked from senior leaders in the region (representatives of the GPs and specialists).  -Senior leaders participated in the process of priority setting for change.  -The commitment of the senior leaders to the project was made visible through their participation in the local steering group. | | | | |

| **Benefits**  **Score** | …discourage patient self-management or system changes.  0 1 2 | …neither encourage nor discourage patient self-management or system changes.  3 4 5 | …encourage patient self-management or system changes.  6 7 8 | …are specifically designed to promote better chronic illness care.  9 10 11 |
| --- | --- | --- | --- | --- |
| **Score 2003:6 Score 2007:9** | | | | |
| -Until 2003 benefits encouraging patients to take up self-management were mainly for patients on insulin therapy (hospital care).  -In 2003 a diabetes passport was introduced for all patients with diabetes (national initiative). One of the benefits linked to the chart was the yearly reimbursement of one hour of dietary advice.  -In October 2004 an individual education program for type 2 diabetes patients was launched in the region. The program was open for all patients on diet and/or oral medication and was free of charge.  -Patients for who starting insulin therapy was considered could benefit of support in primary care (self-monitoring and education). | | | | |

**Part 2: Community Linkages.** Linkages between the health delivery system (or provider practice) and community resources play important roles in the management of chronic illness.

| **Components** | **Level D** | **Level C** | **Level B** | **Level A** |
| --- | --- | --- | --- | --- |
| **Linking Patients to Outside Resources** Score | …is not done systematically.  0 1 2 | …is limited to a list of identified community resources in an accessible format.  3 4 5 | …is accomplished through a designated staff person or resource responsible for ensuring providers and patients make maximum use of community resources.  6 7 8 | … is accomplished through active coordination between the health system, community service agencies and patients.  9 10 11 |
| **Score 2003:1 Score 2007:5** | | | | |
| -Patients attending the education programme were informed about outside resources, e.g. the local diabetes patient organisation, the insurance companies,.. | | | | |

| **Partnerships with Community Organizations** Score | …do not exist.  0 1 2 | …are being considered but have not yet been implemented.  3 4 5 | …are formed to develop supportive programs and policies.  6 7 8 | …are actively sought to develop formal supportive programs and policies across the entire system.  9 10 11 |
| --- | --- | --- | --- | --- |
| **Score 2003:0 Score 2007:6** | | | | |
| -Partnerships with community organisations were established e.g. information sessions for patients were organized in collaboration with the local diabetes patient organisation; training sessions were organized for staff in the local nursing homes; information sessions were organized for senior organisations; … | | | | |

| Regional Health PlansScore | …do not coordinate chronic illness guidelines, measures or care resources at the practice level.  0 1 2 | …would consider some degree of coordination of guidelines, measures or care resources at the practice level but have not yet implemented changes.  3 4 5 | …currently coordinate guidelines, measures or care resources in one or two chronic illness areas.  6 7 8 | …currently coordinate chronic illness guidelines, measures and resources at the practice level for most chronic illnesses.  9 10 11 |
| --- | --- | --- | --- | --- |
| **Score 2003:0 Score 2007:6** | | | | |
| -The local steering group coordinated guidelines and measures for type 2 diabetes in the region, in consultation with the interdisciplinary study group.  -There was no coordination of resources. Extra services introduced during the project (e.g. appointment of a program manager, establishment of an education program) were financed with project funding, other regular services (visits with the GP, specialist, ..) were financed, as before, on a fee-for-service base. | | | | |

**Part 3: Practice Level.** Several components that manifest themselves at the level of the individual provider practice (e.g. individual clinic) have been shown to improve chronic illness care. These characteristics fall into general areas of self-management support, delivery system design issues that directly affect the practice, decision support, and clinical information systems.

-------------------------------------------------------------------------------------------------------------------------------------------------------------

**Part 3a: Self-Management Support.** Effective self-management support can help patients and families cope with the challenges of living with and treating chronic illness and reduce complications and symptoms.

| **Components** | **Level D** | **Level C** | **Level B** | **Level A** |
| --- | --- | --- | --- | --- |
| **Assessment and Documentation of Self-Management Needs and Activities** Score | …are not done.  0 1 2 | …are expected.  3 4 5 | …are completed in a standardized manner.  6 7 8 | …are regularly assessed and recorded in standardized form linked to a treatment plan available to practice and patients.  9 10 11 |
| **Score 2003:3 Score 2007:6** | | | | |
| -Assessment and documentation of self-management needs and activities was recommended in the guideline type 2 diabetes distributed among all GPs in 1997 and 2005 (national initiative).  -In a survey at the start of the project the majority of care providers mentioned to assess self-management needs and activities on an ad hoc basis.  -Among patients attending the education program the self-management needs and activities were assessed in a standardized way. Participation in the program increased gradually. Two years after introduction of the program about 20% of the patients on diet or oral therapy attended the program. | | | | |
|  | | | | |

| **Self-Management Support**  **Score** | …is limited to the distribution of information (pamphlets, booklets).  0 1 2 | …is available by referral to self-management classes or educators.  3 4 5 | …is provided by trained clinical educators who are designated to do self-management support, affiliated with each practice, and see patients on referral.  6 7 8 | …is provided by clinical educators affiliated with each practice, trained in patient empowerment and problem-solving methodologies, and see most patients with chronic illness.  9 10 11 |
| --- | --- | --- | --- | --- |
| **Score 2003:1 Score 2007:6** | | | | |
| -Self-management support was recommended in the guideline type 2 diabetes distributed among all GPs in 1997 and 2005 (national initiative). In a survey at the start of the project the majority of care providers mentioned to give information about different aspects of diabetes. Some are using pamphlets or booklets.  -During the project an education program, based on the empowerment theory, was established in the region. Trained diabetes educators provided the education program. Two years after the introduction of the program 70% of the GPs had referred at least one patient to the program. About 20% of the patients on diet or oral therapy attended the program. | | | | |

| **Addressing Concerns of Patients and Families** Score | …is not consistently done.  0 1 2 | …is provided for specific patients and families through referral.  3 4 5 | …is encouraged, and peer support, groups, and mentoring programs are available.  6 7 8 | …is an integral part of care and includes systematic assessment and routine involvement in peer support, groups or mentoring programs.  9 10 11 |
| --- | --- | --- | --- | --- |
| **Score 2003:1 Score 2007:6** | | | | |
| -Addressing concerns of patients and families was recommended in the guideline type 2 diabetes distributed among all GPs in 1997 and 2005 (national initiative). In a survey at the start of the project the majority of care providers mentioned to address the concerns of their patients on an ad hoc basis.  -Among patients attending the education program concerns were assessed in a standardized way. Their relatives often accompanied patients. There was no specific program targeted to the family. Participation in the program increased gradually. Two years after introduction about 20% of the patients on diet or oral therapy attended the program. | | | | |

| **Effective Behavior Change Interventions and Peer Support**  **Score** | …are not available.  0 1 2 | …are limited to the distribution of pamphlets, booklets or other written information.  3 4 5 | …are available only by referral to specialized centers staffed by trained personnel.  6 7 8 | …are readily available and an integral part of routine care.  9 10 11 |
| --- | --- | --- | --- | --- |
| **Score 2003:0 Score 2007:7** | | | | |
| -In 2003 there was no structured education program available in primary care. Until 2004 structured education was limited to patients on insulin therapy treated in the hospital setting.  -During the project a structured education program was established in primary care. Group education sessions were piloted in the region. Two years after introduction about 20% of the patients on diet or oral therapy attended the program. | | | | |

## Part 3b: Decision Support. Effective chronic illness management programs assure that providers have access to evidence-based information necessary to care for patients--decision support. This includes evidence-based practice guidelines or protocols, specialty consultation, provider education, and activating patients to make provider teams aware of effective therapies.

| **Components** | **Level D** | **Level C** | **Level B** | **Level A** |
| --- | --- | --- | --- | --- |
| Evidence-Based Guidelines Score | …are not available.  0 1 2 | …are available but are not integrated into care delivery.  3 4 5 | …are available and supported by provider education.  6 7 8 | …are available, supported by provider education and integrated into care through reminders and other proven provider behavior change methods.  9 10 11 |
| Score 2003:3 Score 2007:8 | | | | |
| -In 1997 a type 2 diabetes guideline was distributed to all GPs (national initiative; adapted version in 2005). The distribution of the guideline was accompanied by an information campaign. In 2003, no specific national actions were planned. Actions in the region were guided by the recommendations in the guideline.  -During the project interdisciplinary training sessions focusing on the implementation of the protocol were organized.  -Audit and feedback were organised in 2004 and 2006. | | | | |

| **Involvement of Specialists in Improving Primary Care** Score | …is primarily through traditional referral.  0 1 2 | …is achieved through specialist leadership to enhance the capacity of the overall system to routinely implement guidelines.  3 4 5 | …includes specialist leadership and designated specialists who provide primary care team training.  6 7 8 | …includes specialist leadership and specialist involvement in improving the care of primary care patients.  9 10 11 |
| --- | --- | --- | --- | --- |
| **Score 2003:2 Score 2007:9** | | | | |
| -In 2003 specialists were involved in the care for type 2 diabetes patients mainly through traditional referral and providing of ex cathedra provider education.  -Specialists were from the start involved in the project. Involvement in the project was made visible through their participation in the local steering group.  -Specialists contributed to different aspects of the project: the development of the protocol, the organization of provider training, the coaching of primary care.  -Specialists were prepared to take up a coaching role, e.g., specialists coached the support program for the initiation of insulin therapy: educators and GPs had the opportunity to consult specialists for advice on the therapy scheme by phone or e-mail.  -At the start the two hospitals were involved equally. Due to a staff switch the participation of one hospital decreased temporary. | | | | |

| **Provider Education for Chronic Illness Care** Score | …is provided sporadically.  0 1 2 | …is provided systematically through traditional methods.  3 4 5 | …is provided using optimal methods (e.g. academic detailing).  6 7 8 | …includes training all practice teams in chronic illness care methods such as population-based management, and self-management support.  9 10 11 |
| --- | --- | --- | --- | --- |
| **Score 2003:2 Score 2007:5** | | | | |
| -In 2003 provider education for chronic illness was provided on an ad hoc basis. Chronic illness and more specific diabetes was a topic of interest. About 80% of the GPs attended at least one training session on diabetes the last 3 years.  -During the project interdisciplinary training sessions focusing on the implementation of the protocol were organized. Sixty percent of the GPs participated in at least one of the training sessions.  -Information about the training sessions was available on the website. Newsletters were sent on a regular basis by e-mail. | | | | |

| **Informing Patients about Guidelines** Score | …is not done.  0 1 2 | …happens on request or through system publications.  3 4 5 | …is done through specific patient education materials for guidelines regarding diabetes type 2  6 7 8 | …includes specific materials developed for patients which describe their role in achieving guideline adherence.  9 10 11 |
| --- | --- | --- | --- | --- |
| **Score 2003:3 Score 2007:7** | | | | |
| -Informing patients about guidelines was recommended in the type 2 diabetes guideline distributed among all GPs in 1997 and 2005 (national initiative). In 2003 a diabetes passport for patients was introduced for all diabetes patients (national initiative). One of the goals of the passport was to involve patients more actively in reaching the targets.  -In a survey at the start of the project the majority of care providers mentioned to inform patients about guidelines on an ad hoc basis.  -The use of the diabetes passport was actively promoted during the project. The study group of GPs and specialists designed a more attractive version of the diabetes passport.  -Among patients attending the education program the guidelines were addressed in a standardized way. | | | | |

## Part 3c: Delivery System Design.

## Evidence suggests that effective chronic illness management involves more than simply adding additional interventions to a current system focused on acute care. It may necessitate changes to the organization of practice that impact provision of care.

In the Begian health care setting teams in primary care are usually flexible and loose networks of single-handed care providers. Most of the care providers work as independent self-employed health professionals.

| **Components** | **Level D** | **Level C** | **Level B** | **Level A** |
| --- | --- | --- | --- | --- |
| **Team Functioning** Score | …is not addressed.  0 1 2 | …is addressed by assuring the availability of individuals with appropriate training in key elements of chronic illness care.  3 4 5 | …is assured by regular team meetings to address guidelines, roles and accountability, and problems in chronic illness care.  6 7 8 | …is assured by teams who meet regularly and have clearly defined roles including patient self-management education, proactive follow-up, and resource coordination and other skills in chronic illness care.  9 10 11 |
| **Score 2003:0 Score 2007:5** | | | | |
| -At the start of the project care providers expressed their need for clear task descriptions and for the creation of a visible network of care providers involved in diabetes care.  -One of the first results of the project was a website with a list of all care providers involved in diabetes care in the region.  -An interdisciplinary care protocol was developed in collaboration with the region. This protocol included clear task descriptions with respect for the role of the GP and guidelines for interdisciplinary communication.  -The task agreements were evaluated but not on a regular basis. In consultation with the region commitment to the protocol was not made visible on the website. | | | | |

| **Team Leadership** Score | …is not recognized locally or by the system.  0 1 2 | …is assumed by the organization to reside in specific organizational roles.  3 4 5 | …is assured by the appointment of a team leader but the role in chronic illness is not defined.  6 7 8 | …is guaranteed by the appointment of a team leader who assures that roles and responsibilities for chronic illness care are clearly defined.  9 10 11 |
| --- | --- | --- | --- | --- |
| **Score 2003:1 Score 2007: 9** | | | | |
| -In health policy documents GPs are often mentioned as the coordinators of the care around the patient. According to the results of the survey at the start of the project and the discussions in the study groups this was not what GPs experienced in the real life setting.  -During the project the role of the GP as the coordinator of the care around the patient was revalued in the interdisciplinary care protocol. The program manager, in consultation with the local steering group and the interdiscipinary study group, evaluated the implementation of the task agreements. | | | | |

| Appointment SystemScore | …can be used to schedule acute care visits, follow-up and preventive visits.  0 1 2 | …assures scheduled follow-up with chronically ill patients.  3 4 5 | …are flexible and can accommodate innovations such as customized visit length or group visits.  6 7 8 | …includes organization of care that facilitates the patient seeing multiple providers in a single visit.  9 10 11 |
| --- | --- | --- | --- | --- |
| **Score 2003:1 Score 2007:2** | | | | |
| -An appointment system was recommended as a way to organize protected time for chronic care in the type 2 diabetes guideline distributed among all GPs in 1997 and 2005 (national initiative). At the start of the project about half of the GPs worked with some kind of appointment system. This system can be used to schedule follow-up in accordance with the guidelines but most of the patients were still seen on an ad hoc basis. There was no (re)call system in place.  -The results of a survey at the end of the project showed a small increase in the percentage of GPs working with an appointment system. | | | | |

| Follow-upScore | …is scheduled by patients or providers in an ad hoc fashion.  0 1 2 | …is scheduled by the practice in accordance with guidelines.  3 4 5 | …is assured by the practice team by monitoring patient utilization.  6 7 8 | …is customized to patient needs, varies in intensity and methodology (phone, in person, email) and assures guideline follow-up.  9 10 11 |
| --- | --- | --- | --- | --- |
| **Score 2003:2 Score 2007:3** | | | | |
| -A follow-up system at regular intervals was recommended in the type 2 diabetes guideline distributed among all GPs in 1997 and 2005 (national initiative) as a way to organize chronic care. In a survey at the start of the project most of the GPs mentioned to see the patients on a regular basis, but nmostly on an ad hoc basis. There was no (re)call system in place.  -The results of a survey at the end of the project showed a small increase in the percentage of GPs working with an appointment system. Some GPs mentioned to pay more attention to scheduling care in accordance tot the guidelines. | | | | |

| **Planned Visits for Chronic Illness Care** Score | …are not used.  0 1 2 | …are occasionally used for complicated patients.  3 4 5 | …are an option for interested patients.  6 7 8 | …are used for all patients and include regular assessment, preventive interventions and attention to self-management support.  9 10 11 |
| --- | --- | --- | --- | --- |
| **Score 2003:0 Score 2007:0** | | | | |
| -The current organization of primary care does not allow organizing planned visits. Most of the practices are small (70% single-handed) and support staff in practice is limited. Only one third of the GPs had some kind of support in practice, often the partner taking up some administrative function. | | | | |
| Continuity of CareScore | …is not a priority.  0 1 2 | …depends on written communication between primary care providers and specialists, case managers or disease management companies.  3 4 5 | …between primary care providers and specialists and other relevant providers is a priority but not implemented systematically.  6 7 8 | …is a high priority and all chronic disease interventions include active coordination between primary care, specialists and other relevant groups.  9 10 11 |
| **Score 2003:4 Score 2007:8** | | | | |
| -Efforts were already made In the past to optimize communication between care providers. In the survey at the start of the project continuity of care was mentioned as an important item for improvement.  -Continuity of care was an important aspect of the interdisciplinary care protocol. Clear recommendations were made about information transfer. | | | | |

**Part 3d: Clinical Information Systems.** Timely, useful information about individual patients and populations of patients with chronic conditions is a critical feature of effective programs, especially those that employ population-based approaches.

| **Components** | **Level D** | **Level C** | **Level B** | **Level A** |
| --- | --- | --- | --- | --- |
| **Registry (list of patients with specific conditions)** Score | …is not available.  0 1 2 | …includes name, diagnosis, contact information and date of last contact either on paper or in a computer database.  3 4 5 | …allows queries to sort sub-populations by clinical priorities.  6 7 8 | …is tied to guidelines which provide prompts and reminders about needed services.  9 10 11 |
| **Score 2003:1 Score 2007:2** | | | | |
| -At the end of the project about 30% of the GPs had a list with the names of patients having diabetes (on paper or on computer). This list was not routinely used to plan follow-up or other actions. | | | | |
| **Reminders to Providers** Score | …are not available.  0 1 2 | … include general notification of the existence of a chronic illness, but does not describe needed services at time of encounter.  3 4 5 | …includes indications of needed service for populations of patients through periodic reporting.  6 7 8 | …includes specific information for the team about guideline adherence at the time of individual patient encounters.  9 10 11 |
| **Score 2003:0 Score 2007:0** | | | | |
| -This option was not available in the software packages currently used by the GPs. | | | | |
| **Feedback**  **Score** | …is not available or is non-specific to the team.  0 1 2 | …is provided at infrequent intervals and is delivered impersonally.  3 4 5 | …occurs at frequent enough intervals to monitor performance and is specific to the team’s population.  6 7 8 | …is timely, specific to the team, routine and personally delivered by a respected opinion leader to improve team performance.  9 10 11 |
| **Score 2003:0 Score 2007:4** | | | | |
| -In 2003 there was no quality monitoring system in place for diabetes patients looked after in primary care.  -In 2004, a regional database with information from approximately 20% of the diabetes population has been set up. Registration of quality indicators from medical records was done by GPs on a paper form. GPs received a compensation for participating in the quality monitoring project.  -The objective of the establishment of a database with yearly feedback was abandoned due to the problems encountered in the registration process. In 2006, a follow-up registration focused only on the patients registered in 2004.  -In 2005, feedback on the quality of care was sent to all GPs and specialists. Reminders linked to the priorities for change were sent on regular basis by e-mail (2005 and 2006). | | | | |
| **Information about Relevant Subgroups of Patients Needing Services**  **Score** | …is not available.  0 1 2 | …can only be obtained with special efforts or additional programming.  3 4 5 | …can be obtained upon request but is not routinely available.  6 7 8 | …is provided routinely to providers to help them deliver planned care.  9 10 11 |
| **Score 2003:0 Score 2007:0** | | | | |
| -This option was not available in the software programs currently used by the GPs. | | | | |

| **Patient Treatment Plans**  **Score** | …are not expected.  0 1 2 | …are achieved through a standardized approach.  3 4 5 | …are established collaboratively and include self management as well as clinical goals.  6 7 8 | …are established collaborative an include self management as well as clinical management. Follow-up occurs and guides care at every point of service.  9 10 11 |
| --- | --- | --- | --- | --- |
| **Score 2003:2 Score 2007:6** | | | | |
| -Patient treatment plans are recommended in the type 2 diabetes guideline distributed among all GPs in 1997 and 2005 (national initiative) as a way to empower patients in achieving targets.  -In 2003 a diabetes passport was introduced for all patients with diabetes (national initiative). One of the purposes of the passport was to enhance the participation of patients in the goal setting. The implementation of the passport in regular care was poor.  -The use of the diabetes passport was actively promoted during the project. Among patients attending the education program the guidelines were addressed in a standardized way and goals were set in collaboration between patient, educator and GP. During the project the study group of GPs and specialists designed a more attractive version of the diabetes passport. | | | | |
